# The association between social relationships and self-harm: a case–control study in Taiwan

**DOI:** 10.1186/1471-244X-13-101

**Published:** 2013-03-26

**Authors:** Chia-Yi Wu, Chin-Kuo Chang, Hui-Chun Huang, Shen-Ing Liu, Robert Stewart

**Affiliations:** 1 Department of Nursing, College of Medicine, National Taiwan University, 1, Section 1, Jen-Ai Road, Taipei, 10051, Taiwan; 2 King’s College London (Institute of Psychiatry), London, UK, De Crespigny Park, London, SE5 8AF, UK; 3 Department of Medical Research, Mackay Memorial Hospital, Taipei, Taiwan, 45, Min-Sheng Road, Tam-Shui, New Taipei City, Taiwan; 4 Mackay Medicine, Nursing and Management College, Taipei, Taiwan, 92, Shengjing Rd., Beitou Dist, Taipei City, 11260, Taiwan; 5 Department of Psychiatry, Mackay Memorial Hospital, Taipei, Taiwan, 45, Min-Sheng Road, Tam-Shui, New Taipei City, Taiwan

**Keywords:** Case–control study, Self-harm, Suicide, Social support, Isolation, Taiwan

## Abstract

**Background:**

Although suicide has been postulated as a result of social breakdown, relatively little attention has been paid to the association between social relationships and non-fatal self-harm. We sought to investigate the extent to which social factors correlate with self-harm in this case–control study.

**Methods:**

The primary outcome was self-harm with hospital presentation. Cases of self-harm from the Emergency Department in a general hospital in Northern Taiwan were recruited, and individually age-and-gender-matched control participants were recruited from non-psychiatric outpatient clinics at the same hospital. The Close Persons Questionnaire was administered and its social support and social network subscales were used to measure social relationships in the 12 months prior to the interview. Other covariates, comprising sociodemographic factors, major life events, physical and mental health, were adjusted in conditional logistic regression models.

**Results:**

A total of 124 case–control pairs were recruited. The mean (standard deviation) age of the case group was 34.7 (12.8) years and 80.6% were female. Higher social isolation score remained significantly associated with self-harm after adjustment (adjusted odds ratio per standard deviation increase 2.92, 95% confidence interval 1.44-5.95) and household size was negatively associated with the outcome (adjusted odds ratio per unit increase 0.54, 95% CI 0.32-0.94).

**Conclusions:**

More limited social networks were associated with self-harm after adjustment for potential confounders. Enhancing social structure and effective networking of people with self-harm to community resources may be important for self-harm management in Asian societies and elsewhere.

## Background

Self-harm is a major public health issue which is substantially more common than completed suicide and a high-risk group for repetition [1-3]. Despite its relatively low prevalence (i.e. 150-500/100,000 [4-6]), self-harm with repetition had significantly raised risk for suicide [2]. Defined as an act of self-injury or self-poisoning with non-fatal outcomes regardless of the intention to die [7], self-harm is a construct differing from completed suicide in terms of the underlying intention of death, the combination of different methods and their lethality, and the prior degree of hopelessness, amongst other distinctions [8]. Suicide on the other hand is a fatal self-inflicted act before which a person may have carried out one or more self-harm acts [9]. Previous research has suggested that the pathways between social rejection or other stress and suicidal ideation may be moderated by social support; however, more understanding is needed of the role of these potentially protective factors [10-13].

Social support, defined in terms of the quality of perceived support from social relationships, was found to confer protection through help-seeking augmentation [14], and was postulated as a rescue factor which moderates stress and suicidal behaviour [12]. However, it has received less research compared to other etiological factors in relation to self-harm. More comprehensive measures of social relationships have been suggested as important if for clarifying the role of social support and social isolation in the complex phenomenon of self-harm [12]. Social support as a broad entity has been defined as including functional (e.g. information, instrumental, practical, emotional support) and structural (e.g. social network, network outside the family, household size) components of support derived from social networks [15]. Measurement strategies seek to characterize both quality and quantity of social support through which social networks may promote health and well-being [15-17]. Although suicide has been postulated as a result of a breakdown in social ties [17], little attention has been paid to social relationships among cases of self-harm. Adequate social support has been found to modify help-seeking behaviour in these groups [14], to regulate personal response to stressors and prevent anxiety or depression [18,19], and to have both direct effects on mental health as well as modifying the negative impact of life events [20,21]. In addition, social networks and interactions in social contacts may affect social functioning and thus the severity of mental illness [22,23]. The degree of help-seeking as well as social relationships has been found to differ between men and women [24]. Thus, we hypothesized that the level of social support and networks would be lower among people with self-harm compared to a control group, and such association would differ by gender after controlling for physical and psychosocial confounders.

Using a sample of cases of self-harm in Taiwan, we sought to investigate the extent to which social relationships associate with self-harm under adjustment for factors recognised to be associated with suicidal behavior [11], including sociodemographic factors and physical and mental health.

## Methods

### Study design and setting

We used a matched case–control study to compare differences in measures of social relationships between people with self-harm and those without. The study was conducted in a teaching hospital in an urbanized area of Taipei in northern Taiwan during August 2005 and July 2006. Ethical approval was acquired from the Research Ethics Committee in the study hospital (Reference No.: MMH-I-S-202). Patients from the Emergency Room (ER) who fulfilled inclusion criteria of the study were approached upon social workers’ referral by fax or the researchers’ active screening of ER daily attendances.

### Participants

The sample of cases was drawn from the ER of the study hospital, while an age-and-gender matched control group was recruited from attendees at an outpatient Family Medicine Department in the same hospital. The principle of matching was based on the principle that degrees of social support and social networks vary by age and gender. Information had been collected on each participant at hospital presentation regarding the self-harm method (for the case group), demographic and psychosocial information, social relationships, health conditions and major life events (described in detail below). The inclusion criteria for the cases were as follows: age over 18 years, clear consciousness and able to make verbal/written communication, and a self-harm act comprising self-poisoning and/or self-injurious behaviour with non-fatal outcome at the index admission, regardless of suicidal intent or personal expectation of a fatal or non-fatal consequence. Those who were severely depressed and unable to cooperate with interviews, who refused to participate or who were unconscious were excluded. The same inclusion/exclusion rules applied to the control group except that controls with any previous self-harm act or contact with psychiatric services were excluded. Specifically, self-poisoning included overdose of prescribed or non-prescribed drugs, ingestion of pesticide or other chemical substances, and deliberate inhalation of gasoline or other evaporates in gas or liquid forms (e.g. carbon-monoxide poisoning by burning charcoal indoors). Self-injury referred to self-harm by any means that can damage to body integrity immediately or as a consequence, including self-cutting, drowning, hanging, self-burning, or jumping from a high place [25].

### Measurements and variables

Cases and controls were compared with respect to the main exposure of social relationships. Potential confounders including physical illness conditions, severity of depression, and major life events. Covariates are described in detail below.

#### Social relationships

The characteristics of social relationships in the 12 months preceding the index date were measured in identical format in cases and controls using the Close Persons Questionnaire (CPQ) [14,26]. Social relationships have typically been defined as two distinct components for the purpose of measurement: namely social support and social network (conceptually equivalent to ‘objective and subjective’ [27] or ‘functional and structural’ [26] respectively). Reliability and validity for the CPQ have been previously established [14,26], and the CPQ itself has been described in detail elsewhere [14], but is briefly summarized below. In evaluating the relationship between study participants and the person nominated as the closest to them, the CPQ social support subscales measure confiding, practical, and negative aspects of the index close relationship. The social network subscales on the other hand measure the degree of involvement in social activities with family/relatives, friends, colleagues, and/or larger social networks reported by the respondents. Three dimensions of social network can be analysed separately: isolation, network beyond the household, and household size. (See the Additional file 1 for more details about scale items and scoring method)

#### Physical and mental health

We recorded self-reported disorders related to every organ system. These disorders were chosen based on common disorders seen in a general hospital. Each disease of a single system was rated 1. The numbers of physical illnesses were summed as an indicator of health conditions, with higher numbers indicating worse physical health. We further double-checked the electronic medical records to confirm each participant’s diagnoses. For mental health evaluation, the Patient Health Questionnaire-9 (PHQ-9) was used to assess depressive symptoms. The nine items on this scale are rated on a 4-point scale, ranging from 0 (never) to 3 (nearly every day) generating a total score ranging from 0 to 27, with higher scores indicating increased likelihood of depressive disorders. The Chinese version of the PHQ-9 has been validated and found to have good reliability and validity [28].

#### Major life events

Participants were asked about the following events and dates of occurrence: personal experience of major illness, injury or assault; death, major illness, injury or assault experienced by a close family member; marital or long-term interpersonal relationship break down; serious dispute with family members, relatives, friends or neighbors; being unemployed for over a month or being made redundant; severe financial crisis; judicial problems to be solved; major material loss; severe behavioural problems of children. Each life event was recorded “1” if present, and total life events were summed to generate an ordinal covariate for statistical analysis.

#### Other covariates

The sociodemographic variables that were adjusted for in the regression models consisted of age, gender, marital status (single, married or cohabitation, divorced/ separated/ widowed), years of formal education, employment status (currently employed, housewife/student/retired, or jobless), and religion (none or any).

### Statistical analysis

The case and control groups were first compared with respect to their demographic characteristics and other covariates. For the purpose of establishing a common platform for comparisons across the components of CPQ, all scores on social support and social networks were standardized by subtracting the control mean and dividing by the control standard deviation (i.e. generating z-scores). Because of the individual matching used in the study design, conditional logistic regression procedures were carried out for both unadjusted and adjusted analyses. Factors that were controlled for in the regression analysis included the sociodemographic measures, number of life events, and physical and mental health. Subgroup analyses were carried out, stratifying by gender. The sample size of the study was chosen based on information from previous literature: taking into account the matched pair design, 124 subjects were required in each comparison group for a minimum detectable relative risk of 1.18 (or 0.85 for protective factors) for the subscale with least effect size (negative aspects of social relationship) at the statistical power level of 85%. SPSS 17.0 for Windows was utilized to perform all the statistical analyses and the significance level (α value) was set at 0.05.

## Results

The physical, psychosocial and demographic characteristics of the 124 cases and 124 age- and gender-matched controls were collected during a one year period and are described in Table 1. The mean age of the cases was 34.7 with a standard deviation of 12.8, and the male-to-female gender ratio was approximately 1:4 (80.6% females). The control sample was matched effectively on these factors. Among the case group, self-poisoning had been carried out by 73.7% and self-injury by 28.3%. The cases were characterized as having a higher proportion of participants with divorced, separated or widowed status of marriage, shorter duration of education, and higher prevalence of unemployment. In terms of social support and network levels, cases had significantly lower levels of self-reported confiding support, practical support, smaller networks beyond the household, and their level of isolation was higher than the controls.

**Table 1:** Comparisons of sociodemographic characteristics, psychosocial conditions, and physical health between groups of cases and matched controls (N = 248)

**Variable**	**Mean ± SD / Number (%)**		
	**Controls**	**Cases**	**p value**^**1**^
	**(n = 124)**	**(n = 124)**	
Age	35.17 ± 13.59	34.73 ± 12.81	--
Gender			
Female	100 (80.65%)	100 (80.65%)	--
Male	24 (19.35%)	24 (19.35%)	
Marital status			<0.05*
Single	68 (54.84%)	63 (50.81%)	
Married/cohabitation	51 (41.13%)	43 (34.68%)	
Divorced/separated/widowed	5 (4.03%)	18 (14.52%)	
Years of education	14.28 ± 3.95	10.90 ± 3.63	<0.01*
Employment status			
Currently employed	81 (65.32%)	66 (53.23%)	
Housewife/student/retired	40 (32.26%)	35 (28.23%)	<0.01*
Jobless	3 (2.42%)	23 (18.55%)	
Religion			
None	73 (58.87%)	73 (58.87%)	0.32
Any	39 (31.45%)	51 (41.13%)	
Social support subscale (CPQ)			
Confiding support	23.39 ± 4.91	21.75 ± 4.20	<0.01*
Practical support	8.10 ± 2.09	7.45 ± 2.30	<0.05*
Negative aspects	7.35 ± 2.36	6.94 ± 2.43	0.17
Social network subscale (CPQ)			
Isolation	1.77 ± 1.22	2.57 ± 1.35	<0.01*
Network beyond the household	10.08 ± 4.31	8.31 ± 3.84	<0.01*
Household size	1.40 ± 0.78	1.40 ± 0.79	0.94
PHQ-9 score	4.28 ± 3.99	14.48 ± 7.07	<0.01*
Number of physical illness	0.86 ± 0.99	1.41 ± 0.79	<0.01*
Number of major life events	0.65 ± 0.98	0.65 ± 0.82	0.99

1 Chi-square tests for categorical variables and paired t-tests for continuous variables.

Abbreviations: Standardised deviation (SD), Close Persons Questionnaire (CPQ), Patient Health Questionnaire (PHQ). * Statistical significance.

In Table 2, associations between z-scored social relationship measures and case/control status were compared using conditional logistic regression models. After adjustment, a strong association persisted between case status and the standardized score of isolation (OR = 2.92; 95% CI: 1.44, 5.95), and a significant negative association was found between self-harm and larger household size (OR = 0.55; 95% CI: 0.32, 0.94). Social support subscale measures were not significantly different between cases and controls after adjustment.

**Table 2:** Crude and adjusted odds ratios of Z-scored subscales of social support / network for self-harm behavior by conditional logistic regressions (N = 248)

	**Odds ratio (95% confidence intervals)**	
**Exposure measures**	**Crude**	**Adjusted^**
Social support subscale (CPQ)		
Confiding support	0.68 (0.52, 0.90)**	0.88 (0.55, 1.41)
Practical support	0.77 (0.61, 0.98)*	0.74 (0.49, 1.09)
Negative aspects	0.85 (0.67, 1.09)	0.69 (0.44, 1.07)
Social network subscale (CPQ)		
Isolation	2.25 (1.58, 3.19)***	2.92 (1.44, 5.95)**
Network beyond the household	0.61 (0.45, 0.83)**	0.75 (0.47, 1.19)
Household size	1.01 (0.79, 1.30)	0.55 (0.32, 0.94)*

^ Adjusted for marital status, years of education, employment status, religion, major life events, and physical/mental illness.

*p <0.05; **p <0.01; ***p <0.001.

Abbreviation: Close Persons Questionnaire (CPQ).

Stratifying primary analyses by gender (Table 3), associations with higher levels of social isolation and smaller household size were only significant in female participants; however, most coefficients did not differ substantially and confidence intervals overlapped, suggesting insufficient evidence for effect modification.

**Table 3:** Gender-specific crude and adjusted odds ratios of Z-scored subscales of social support / network for self-harm behavior by conditional logistic regressions (N = 248)

	**Odds ratio (95% confidence intervals) between z-scored exposures and self-harm**			
	**Crude**		**Adjusted^**	
**Measures**	**Female (n = 200)**	**Male (n = 48)**	**Female (n = 200)**	**Male (n = 48)**
Social support subscale (CPQ)				
Confiding support	0.63 (0.46, 0.85)**	1.05 (0.54, 2.02)	0.82 (0.50, 1.35)	2.65 (0.02, 417.50)
Practical support	0.66 (0.49, 0.87)**	1.45 (0.80, 2.61)	0.69 (0.45, 1.06)	2.42 (0.04, 150.72)
Negative aspects	0.83 (0.64, 1.08)	0.98 (0.52, 1.83)	0.73 (0.47, 1.15)	0.21 (0.01, 38.86)
Social network subscale (CPQ)				
Isolation	2.21 (1.52, 3.20)***	2.53 (0.88, 7.29)	2.58 (1.28, 5.21)**	11.40 (0.03, 4367.30)
Network beyond the household	0.60 (0.43, 0.83)**	0.68 (0.33, 1.41)	0.82 (0.51, 1.30)	0.24 (0.01, 69.25)
Household size	1.03 (0.78, 1.36)	0.95 (0.54, 1.67)	0.55 (0.32, 0.97)*	0.82 (0.01, 65.36)

^ Adjusted for marital status, years of education, employment status, religion, major life events, and physical/mental illness conditions.

* p < 0.05; **p < 0.001; ***p <0.001.

Abbreviations: Close Persons Questionnaire (CPQ), Patient Health Questionnaire (PHQ).

## Discussion

In this sample of people who accessed emergency care after self-harm, social networks and household size were found to be more limited and smaller compared to a hospital control group matched on age and gender. Our findings supported the view that prior structural social support has more influence on self-harm than the functional social support provided by the nominated closest person to the participants [29-31]. The results implied that the importance of networking from family and friends was more than that of perceived support from the closest person, in relation to self-harm within this East Asian society.

Reasons for social isolation include sociodemographic changes such as ageing and unemployment, loss of a partner, isolated living environment, and societal position [32], and isolation may also be caused by physical and mental disorders or arise after major life events [33]. In this analysis we controlled for a range of potential confounders including physical and mental health. It might be that, given the resource flow and social support derived from the social networks, it was the structural conditions and quantity of support that determined individual adaptive behaviour as suggested by Berkman and colleagues [16]. In other words, the quantity of social relationships might be more influential in self-harm aetiology than the quality of support received through specific networks. It might also be that, within the Taiwan context, alternative social networks may dilute the “no escape” type of thought in the process when one formulates suicidal ideation or self-harm plans [12] or may form a pathway towards support in need for people experiencing suicidal crisis [34]. The results add to the previous model in suggesting that social isolation or rejection could play a major role in the pathway to suicidal risk, with a lesser influence of perceived/ functional social support [12]. However, further research is required to confirm this relationship.

Findings from randomized controlled trials have suggested that increasing personal access to peer support may result in some reduction of self-harm repetition or suicide ideation [35,36]. For example, short-term periodic telephone contacts and networking from trained family or friends nominated by young women with self-harm and premorbid mood-related symptoms may decrease suicide ideation and attempts [36]. Our observational findings supported this and suggest a need for further evaluation of interventions to improve networking for people at risk of self-harm.

Strengths of our study include the relatively large case and control groups compared to previous studies [37-39] and a relatively comprehensive social support and network measurement with a multidimensional approach. However, case control studies are recognized to be methodologically challenging and inferences should be drawn with caution. The fact that both case and control participants were sampled from a single general hospital in an urban district of Taipei may limit generalizability and findings require replication in other settings. Hospital controls might also lead to under-estimated associations of interest because of excess morbidity and physical disability associated with smaller social networks. A larger sample size would have increased statistical power to detect differences and negative findings should be interpreted with caution. Further, more specific matching of the control group could have taken place (for example with respect to employment, education, marital status and residence) as a means of adjustment; however, over-matching creates potential problems for case control studies, in addition to reducing the feasibility of a study where there are too many groups requiring individual matching; therefore we took a relatively parsimonious approach and adjusted for other potential confounding factors in regression models. Reverse causality, a related issue, also cannot be excluded in a case control design. For ethical and logistical reasons, cases could not be interviewed immediately after the index self-harm episode and it is possible that a subsequent change in social relationships followed on from the self-harm which was being rated when the CPQ was carried out (despite instructions to the contrary).

The study has implications for mental health practice and policy makers. In an open referral system such as that operating in Taiwan’s health service, social network plays an important potential protective role through the availability of others to support, listen, and respond to people at risk. Network members in the lay or healthcare system may also act to encourage prompt medical review and thus reduce self-harm risk through indirect means. Although interventions have yet to be developed with demonstrable efficacy in relation to preventing self-harm acts [40], recent studies have suggested the need for psychiatric and social approaches to reduce isolation, especially in middle-age [41]. Future studies could explore the needs of isolated groups and pilot strategies to improve living circumstances, as well as investigate the benefits of targeting high risk groups for enhanced social networks. Replication of the findings in this and similar cultural context is also required, as is prospective longitudinal research.

## Conclusions

More limited social networks were associated with self-harm after adjustment for potential confounders. Enhanced social structure and raising awareness of networking people with self-harm to community resources may be important for self-harm management in Asian societies and elsewhere.

## Consent

Written informed consent was obtained from the patient for publication of this report and any accompanying images.

## Competing interests

The author(s) declare that they have no competing interests.

## Authors’ contributions

CW contributed in study concept and design, acquisition of partial data, data interpretation, drafting and submission of the manuscript. SL had been involved in acquisition of funding, manuscript revision and provided ideas during the study procedure. HH was responsible for data collection and study design. CC participated in the design of the study, carried out statistical analysis, and provided opinions towards the manuscript. RS participated in draft revision and provided critical contents to the manuscript. All authors read and approved the final manuscript.

## Authors’ information

CW is currently a nursing lecturer and epidemiological researcher who has published papers on topics related to suicide prevention and help-seeking behaviour among people with self-harm. SL is a visiting psychiatrist who has broad publications regarding people with major psychiatric disorders including alcohol use disorders, suicidal behaviour and self-harm, depressive and anxiety disorders. HH is the leader of research assistants in the research team and has been involved in psychiatric field studies for more than a decade. CC is an epidemiologist and senior researcher at the Institute of Psychiatry who excels in study design and statistical analysis relating to the field of oncology and psychiatry. RS is Professor of Psychiatric Epidemiology and Clinical Informatics at King’s College London Institute of Psychiatry with interests in late-life mental health and clinical data resources, in addition to psychosomatics including self-harm and suicide.

## Pre-publication history

The pre-publication history for this paper can be accessed here:

http://www.biomedcentral.com/1471-244X/13/101/prepub

## Supplementary Material

Additional file 1: Appendix Click here for file
