# Supplementary material for: The association between social relationships and self-harm: a case–control study in Taiwan
Source: BMC Psychiatry. 2013 Mar 26;13:101. doi: 10.1186/1471-244X-13-101 (PMC3621841; doi:10.1186/1471-244X-13-101)
Supplement: Additional file 1: Appendix [file 1471-244X-13-101-S1.docx]

Appendix

THE CLOSE PERSONS’ QUESTIONNAIRE (CPQ)

**The Social Support Subscales**

The ratings for the items: Not at all (0), a little bit (1), quite a few (2), a great deal (3).

1) How much, in the last 12 months … did this person give you information, suggestions and guidance that you found helpful?

2) How much, in the last 12 months … could you rely on this person (was this person there when you needed him/her)?

3) How much, in the last 12 months … did this person make you feel good about yourself?

4) How much, in the last 12 months … did you share interests, hobbies and fun with this person?

5) How much, in the last 12 months … did this person give you worries, problems and stress?

6) How much, in the last 12 months … did you want to confide in this person?

7) How much, in the last 12 months … did you confide in this person?

8) How much, in the last 12 months … did you trust this person with

your most personal worries and problems?

9) How much, in the last 12 months … would you have liked to have

confided more in this person?

10) How much, in the last 12 months … did talking to this person makes things worse?

11) How much, in the last 12 months … did he/she talk about his/her personal worries with you?

12) How much, in the last 12 months … did you need practical help with this person with major things (e.g. to look after you when ill, help with finances, children)?

13) How much, in the last 12 months … did this person give you practical help with major things?

14) How much, in the last 12 months … would you have liked more practical help with major things from this person?

15) How much, in the last 12 months … did this person give you practical help with small things when you needed it (e.g. chores, shopping, watering plants, etc.)?

The scoring method for the CPQ Social Support subscales:

1. **Confiding/emotional support scale** = sum of scores for Items 1, 2, 3, 4, 6, 7, 8 and 11
2. **Practical support scale** = sum of questions 12, 13 and 15
3. **Negative aspects of support scale** = sum of scores for questions 5, 9, 10 and 14

**The Social Network Subscales**

Social contact frequency ratings (for Items 1, 3, 4, 6, and 8): Almost daily (1), about once a week (2), about once a month (3), once very few months (4), never or almost never (5).

Social networking scale ratings (for Items 2 and 5): None (1), 1-2 (2), 3-5 (3), 6-10 (4), more than 10 (5).

Social organizations belonging (for Item 7): Yes (1), No (2).

1) Are there any relatives outside your household who you regularly visit or who visit you?

2) How many relatives do you see once a month or more?

3) How often do you ever see anyone from work, socially out of work hours?

4) Do you have any friends or acquaintances you visit or who visit you? (Not necessarily the same person each time).

5) How many friends or acquaintances do you see once a month or more?

6) How often do you attend religious services (apart from weddings and funerals)?

7) Do you belong to any clubs or organisations (social or recreational groups, trade union, commercial groups, professional organisations, political parties, sports clubs, cultural groups, pressure groups, etc)? (If YES, answer the following question)

8) Taking all of these activities together, how often do you attend clubs or organisations?

9) Does anyone live in your household besides you? (If YES, answer the following question)

10) Who lives in your household besides you? Spouse or partner, mother, father, spouse’s father, spouse’s mother (score 1=Yes, 2=No to each of the above questions), Children under 5, Children aged 5 – 15, Children over 15, and any other people (write down number for each of the above questions)

The scoring method for the CPQ Social Network subscales:

**A. Isolation Scale (scored 0 – 5)**

Score 1 for each possible area of social contacts if no contacts as follows: Household (score 1 if nobody living in household), relatives (score 1 if Item 1 = 5, or Item 2 = 1), work (score 1 if Item 3 = 5), friends (score 1 if Item 4 = 5 or Item 5 = 1), religion (score 1 if Item 6 = 5), social organizations (score 1 if Item 7 = 2 or if Item 8=5).

**B. Network beyond the household scale (scored 0 – 28)**

First, score Item 8 as 5, if 7 = 2

Next, rescore the following questions as 0-4 (instead of 1-5): Items 1, 2, 3, 4, 5, 6, 8

Reverse the scoring for Items 1, 3, 4, 6 and 8

**C. Household Size Scale (scored 0 – 2)**: For Item 10, score 0 if no other adults living in the household, score 1 if sharing household with one other adult, score 2 if living with two or more other adults.
